# Predictive Modeling of Long-Term Survivors with Stage IV Breast Cancer Using the SEER-Medicare Dataset

**DOI:** 10.3390/cancers16234033

**Published:** 2024-12-01

**Authors:** Nabil Adam, Robert Wieder

**Affiliations:** 1 Phalcon, LLC, Manhasset, NY 11030, USA; n.adam@phalconllc.com; 2 Newark Campus, Rutgers University, Newark, NJ 07102, USA; 3 Rutgers New Jersey Medical School, Newark, NJ 07103, USA; 4 Rutgers Cancer Institute of New Jersey, Newark, NJ 07103, USA

**Keywords:** deep learning, breast cancer, overdiagnosis and overtreatment, SEER-Medicare-linked dataset

## Abstract

Importance: Treatment of women with stage IV breast cancer (BC) extends population-averaged survival by only a few months. Here, we develop a model for identifying individual circumstances where appropriate therapy will extend survival while minimizing adverse events. Objective: Our goal is to develop high-confidence deep learning (DL) models to predict survival in individual stage IV breast cancer patients based on their unique circumstances generated by patient, cancer, treatment, and adverse event variables. We previously showed that predictive DL survival modeling of potentially curable stage I–III patients can be improved by combining time-fixed and time-varying covariates. Here, we demonstrate that DL-based predictive survival modeling in stage IV patients, where treatment does not offer a cure, can generate accurate individual survival predictions by considering subsequent lines of potential treatment to guide therapy. This guidance is rarely obtainable in the nearly limitless scenarios of metastatic disease. Design, Setting, and Participants: We applied the SEER-Medicare linked dataset from 1991 to 2016 to investigate 14,312 unique stage IV patients with 1,880,153 entries. We used DeepSurv- and DeepHit-, Nnet-survival- and Cox-Time DL-based predictive models to consider the combination of time-fixed and time-varying covariates at each visit for each patient. We adopted random sampling to divide the input dataset into training, validation, and testing sets. We verified the models’ implementation using the pycox package and fine-tuned the models using the open-source library Amazon SageMaker Python SDK 2.232.2 (software development kit). Our results demonstrated the proof of principle of the models by generating individual patients’ survival curves. Conclusions and Relevance: By extending the survival prediction models to consider stage IV BC patients’ time-fixed and time-varying covariates, we achieved a prediction error below 10%. Based on their circumstance-specific situations, these models can predict survival in individual stage IV patients with high confidence. The models will serve as an important adjunct to treatment decisions in patients with stage IV BC and test what-if scenarios of treatment or no treatment options to optimize therapy for extending patient lives and minimizing adverse events.

## Simple Summary

Most patients with stage IV breast cancer cannot be cured; hence, the goal of therapy is to prolong survival while maintaining a good quality of life. Guidance for treating patients in the nearly limitless scenarios after disease progression following the first few lines of therapy comes from clinical trials conducted in patients who have had limited treatment and do not address these many complex scenarios. We used a form of artificial intelligence called deep learning to investigate patients with stage IV breast cancer from the SEER-Medicare dataset to develop predictive modeling in individual patients. We achieved approximately 90% accuracy in survival predictions in individual patients by combining features from the patient, the cancer, their treatments, and adverse events from treatment. These models can guide decision-making regarding patients’ treatment, where guidance is unavailable, to extend their lives while maintaining a good quality of life.

## 1. Introduction

A total of 43,170 women died from breast cancer (BC) in the USA in 2023, most from stage IV disease [1,2,3,4,5]. Despite some progress in prolonging survival, their overall median survival remains 2–3 years [3,4]. Treating patients with stage IV BC can prolong the population’s average survival by only a few months [6,7,8]. Systemic treatment options vary in the wide range of scenarios generated by patient- and cancer-related variables and include many possible options from combinations of chemo-, hormone-, bio-, or immune-therapy categories [4,9,10].

However, despite the grim overall numbers, some long-term survivors exist [11,12]. There are several variables linked to survival times. A key variable includes the presentation of the disease. Patients with de novo presentation of stage IV BC have a median survival superior to that of patients with recurrent disease [13,14,15,16,17,18,19]. In one study, the median survival of patients with de novo metastatic BC was 41 months, while the median survival of patients with recurrent BC was 25 months [17]. In patients with recurrent disease, shorter metastasis-free intervals of <24 months are associated with worse survival than longer metastasis-free intervals [14,16,18,19]. Neoadjuvant treatment for the original local disease had a significant positive association with survival [20].

In patients with de novo diagnosis of stage IV disease, risk stratification models identified variables at the time of diagnosis that were positively and negatively associated with overall survival. These variables included age at diagnosis, particularly age over 80 years, premenopausal status, Karnofsky performance status, estrogen receptor (ER), progesterone receptor (PR) and human epidermal growth factor receptor 2 (Her2) status, tumor histology, grade, tumor size categories 0–3 vs. 4, lymph node status, number of metastatic organ systems from 1 to 4, number of metastases, number of metastatic sites, bone-only metastases +/−, visceral metastases +/−, brain-only metastases +/−, frontline therapy with Her2 antibody or hormone blockade, and combinations of these variables [14,17,21,22,23,24,25,26,27,28].

In addition to these time-fixed variables, time-varying variables occurring after initial diagnosis of de novo stage IV disease are predictive of survival. These include resection of the primary tumor [29,30,31,32], of locoregional metastases [33], or of lung-only metastases [34]. The impact of primary tumor resection on survival is affected by age, race, cohabitation status, income, tumor size, grade and histology, ER, PR or Her2 status, expression of Ki_67_ and CA_15_, alkaline phosphatase, lymphovascular invasion, lymph node status, metastasis to brain, liver, and lung, and chemotherapy [27,29,30].

With respect to systemic therapy, although population-averaged survival extension is modest, multiple treatment- and circumstance-associated combinations can result in prolonged survival in specific cases. Systemic treatment options consist of individual or combinations of categories of chemo-, hormone-, bio-, or immune-therapy [4,9,10]. Variables that impact survival include treatment timing, dosing intensity and density, duration, sequential or intermittent drug administration, alternating modalities, first or subsequent lines of treatment [11,12], and adverse events (AEs) [9], particularly in association with other negative patient risk factors [35,36,37,38,39,40,41,42]. For example, long-term survival of Her2+ patients with Her2-targeted therapy, taxane-based, hormone maintenance therapy, nab-paclitaxel therapy, or multimodality therapy, with or without local treatment, was dependent on hormone receptor expression, disease burden, soft tissue or bone vs. visceral metastases, resection of the primary tumor or metastases and young age [43,44,45,46,47,48,49], and on freedom from disease progression after a year of therapy [50].

Overall, the highest responses in stage IV BC are observed with the first line of therapy, followed by rapidly diminishing returns [11,12,42]. Disease progression after the first few treatment regimens results in exceptionally heterogeneous disease, with cancer cells and tumor stroma selected for treatment resistance and an enhanced capacity to re-metastasize [51]. Decisions to treat to prolong survival also include considerations of treatment-induced AEs [52,53,54,55,56,57,58,59,60,61]. Guidance from clinical trials for the many heterogeneous scenarios of later lines of therapy is not available. Therefore, it would be highly beneficial to patients and their caregivers to know the patient- and cancer-specific circumstances where a patient could expect survival benefits from specific treatments. Population-based predictive normograms achieved concordance indices (C-index) below 0.700 using significant univariate risk factors [17,62], which rose to 0.737 using Cox regression modeling of five prognostic categories [63]. However, predictive models for individual patient survival are needed to navigate the exceptionally large number of potential circumstance-specific scenarios where treatment may prolong life and minimize adverse events.

Given the rich and variable population-based predictive data with significant variation and the generally limited survival with stage IV BC, we have undertaken a deep learning (DL) (a subfield of artificial intelligence) approach to generate predictive modeling of individual patient survival from the SEER-Medicare data of time-fixed and time-varying covariates. Using this unique approach, we can identify patient and treatment scenarios that will result in long-term survival. The models will help caregivers identify specific treatments from a wide variety of choices that will optimize survival in individual patients and unique circumstances.

## 2. Methods

### 2.1. Study Data: SEER-Medicare-Linked Dataset

Unlike the SEER dataset, which does not provide data on cancer therapy, the SEER-Medicare (S-M)-linked dataset created almost 30 years ago is considered a major source to assess cancer care and outcomes in the US [64]. The S-M-linked dataset provides information about Medicare beneficiaries with cancer. This dataset includes cancer incidence data for about 26% of the US population in various regions. Medicare data include 883,053 patients from 1991 to 2015 and are organized into a SEER cancer file, a MEDPAR file, an Outpatient file, an NCH file, and the CCflag file [65]. The number of cancer patients and their corresponding records in each file were outlined previously [65]. We fused the data in the various files using the Observational Medical Outcomes Partnership (OMOP) common data model [66] and computed the stage IV patients’ time-varying comorbidity index, as before [67]. The patient numbers in each file reflect the fact that some patients recorded encounters in multiple files. This enabled us to transform data contained within those disparate files into a common format (data model) as well as a common representation (terminologies, vocabularies, coding schemes).

### 2.2. Inclusion Criteria

In our study, we included women with the diagnosis of stage IV BC, with no prior malignancies, and included all recorded comorbidities, treatments, patient variables, and cancer variables. A patient must have been enrolled, for age eligibility, in Medicare Parts A and B with no HMO enrollment 1 month before BC diagnosis. Table 1 includes the number of stage IV patients, their number of entries, mean age, and comorbidity index.

### 2.3. Data Cleaning and Standardization

Data cleaning, transformation, standardization, and bucketing for continuous values, e.g., age and comorbidity index, were performed to eliminate duplicates or invalid data and ensure all features were numeric and standardized [65].

**Figure 1:**
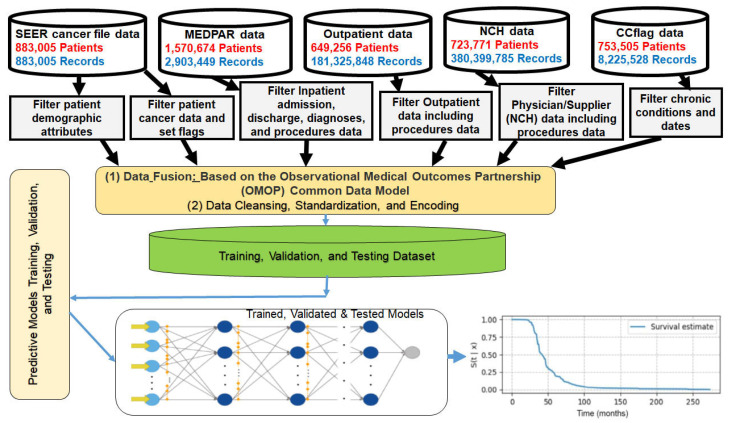
Dataset distribution and model development.

Figure 1 depicts a flow diagram of the dataset distribution used for model development and testing.

### 2.4. Treatments and Adverse Events

We categorized all ICD-9 codes of potential adverse events into 18 categories representing general categories of adverse cancer treatment events included in the literature and classified, by mechanisms of action, 141 HCPCS drug J codes into 82 chemotherapy drugs, 49 biotherapy, and 10 hormone therapy, as before [68].

### 2.5. Predictive Modeling

#### 2.5.1. Discrete Time-to-Event Data

Patients’ visits occur at discrete times, e.g., on a given day, month, or year, with irregular elapsed time between two consecutive visits. On a given visit, the patient’s survival status is recorded. We accounted for a patient’s time-fixed covariates (e.g., race) and time-varying covariates (e.g., treatments administered and adverse events experienced at this and earlier visits) [67].

In our dataset, we have 14,312 BC stage IV patients and 1,880,153 entries over the period 1991–2015. Since we selected patients with ICD-9 diagnosis codes for this study, the study period was set to 1991–2015. Thus, our study is right-censored, i.e., some patients are not followed all the way to their event (death) time, resulting in censored time (31 December 2015) instead of event time. Thus, instead of observing the event (death in our case) at time, $T^{*}$, we observe a possibly right-censored event time, $T=\min\left\{T^{*},\;C^{*}\right\},\;\mathrm{where}\;C^{*}$ is the censoring time (31 December 2015 in our case). We also observe the indicator $D=1\{T=T^{*}\}$ labeling the observed event time as an event or a censored observation.

The general idea of prediction using this discrete-time framework is to build models predicting the likelihood of surviving each visit. In our case, the event of interest is death. Let $T^{*}$ be the time to death since diagnosis, and $T=\min\left\{T^{*},\;C^{*}\right\},\;\mathrm{where}\;C^{*}\;$ is the censoring time, and x is a covariate vector. We are predicting the probability of a patient experiencing failure (death) by time t. This probability, referred to as the cumulative incidence function, is given by $P\left(T\leq t|x\right).$ An alternative to this probability is the survival function, $S\left(t\right)=P\left(T>t|x\right),$ and the hazard rate, $h\left(t|x\right).$ The Cox proportional hazard (CPH) model [69] is the most used model in survival analysis. It provides a semi-parametric hazard rate of $h\left(t|x\right),$ which is the product of a baseline hazard, $h_{0}\left(t\right),$ and a relative risk function, $e^{g(x)}$, i.e., given by $h\left(t|x\right)=h_{0}\left(t\right)e^{g(x)},\;g\left(x\right)=\beta^{T}x$, where x is a covariate vector, and β is a parameter vector. The proportionality hazards assumption, i.e., the effect of a given covariate of a patient remains constant over time, is not realistic in most clinical scenarios [70] and rarely has a real justification [71].

We applied the existing DL-based models DeepSurv [72] to extend the Cox regression models with neural network estimators [73,74], and DeepHit [75], Nnet-survival [70], and Cox-Time [76]. Unlike DeeSurv, developed upon the Cox proportional assumption, the DeepHit, Nnet-survival, and Cox-Time models do not make such an assumption.

#### 2.5.2. Time-Varying Covariates

We extended a covariate$,\;x_{i}$, of a patient, i, to include time-fixed covariates together with time-varying covariates that summarize the patient’s history from previous visits [67]. The models, thus, generate a realistic prediction of the patient’s survival by taking into account not only the patient and cancer variables at the time of diagnosis but also subsequent events associated with treatments and patient’s age and health status. This enables us to predict associations between specific treatments in specific scenarios and outcomes relating to quality of life, progression-free survival, and survival in the spectrum of stage IV cancer settings.

### 2.6. Experiments

#### 2.6.1. Data Preparation

Our data consisted of patient-level records with a single row for each patient and a column for each patient covariate as described above. A patient had a column indicating the corresponding value of $T=\min\left\{T^{*},\;C^{*}\right\},\;$ and a column indicating the patient event status at that time. The time horizon (1 January 1991 through 31 December 2015) was divided into months.

Our model provides an estimate of a patient’s complete survival curve, S(*t*); these estimated survival curves represent event probabilities as time functions.

#### 2.6.2. Performance Metrics

In general, metrics for measuring the model’s predictive performance are often in terms of discrimination, i.e., the model’s ability to distinguish between patients with a high and low risk of experiencing the event, and calibration, i.e., the agreement between the estimated and actual incidence of the event [77].

At time 0, patients are event-free, and their survival status changes from one time period to another. Thus, performance metrics for assessing the predictive accuracy of time-to-event outcomes are time-dependent. In addition to accounting for time, performance metrics must also account for censoring. Two such metrics that are commonly used to measure the predictive accuracy of survival prediction models are (1) time-dependent concordance index [78], and (2) time-dependent Brier score [79,80,81]. The concordance index, or C-index, referred to as the AUC (area under the receiver operating characteristics curve), is the most commonly used method to measure the model’s predictive accuracy. It estimates the probability that, for two randomly selected patients, the predicted survival times of the two patients have the same ordering as their true survival times [82]. The C-index is a scale-free measure with 1 representing perfect discrimination and 0.5 representing discriminative ability similar to chance. Brier score, the predictive error, assesses the model’s calibration and discrimination ability, with lower values indicating better prediction. Similar to Kvamme [76], we use the time-dependent C-index by Antolini et al. [78] and use the integrated Brier score (IBS) by Graf et al. [80] distribution.

#### 2.6.3. Hyperparameter Tuning

**Figure 2:**
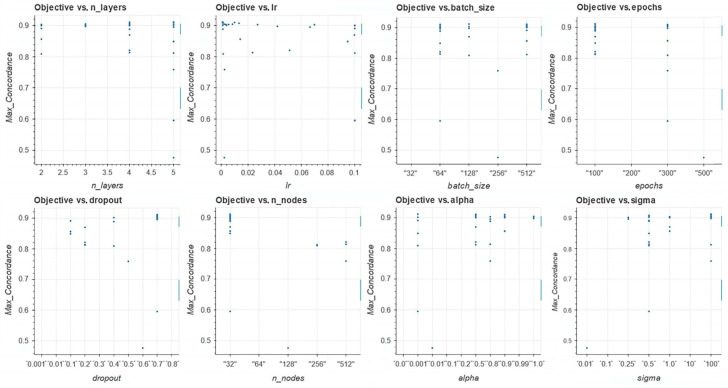
Concordance vs. model hyperparameters: n-layers, Ir, batch size, epochs, dropout, n_nodes, alpha and sigma.

To fine-tune the models, i.e., identify optimal values of the network’s hyperparameters, we used the open-source library Amazon SageMaker Python SDK 2.232.2 (software development kit). Table 2 includes the list of the hyperparameters and their ranges. The experiments were conducted using five-fold cross-validation using a Bayesian optimization search scheme [83]. The Bayesian optimization scheme has been shown to outperform other state-of-the-art global optimization algorithms on a number of challenging optimization benchmark functions [84]. Figure 2 shows the time-dependent concordance (performance metric) for each model and its corresponding hyperparameters.

#### 2.6.4. Validation

To validate our implementation using the pycox package, we applied DeepHit, Nnet-survival, and Cox-Time to the real datasets METABRIC [85] and SUPPORT [86] and conducted the experiments using five-fold cross-validation. As we demonstrated before [67], the published results of each of these models using the Metabric and SUPPORT datasets are within our results’ 95% confidence level, respectively (we used only the time-dependent C-index as the performance metric) [87].

## 3. Results

### 3.1. Improved Predictive Capacity When Adding Time-Variable Covariates to Time Fixed Covariates

Our results, included in Table 3, demonstrated significant improvement in concordance with the models using the extended patient covariates compared to the time-fixed covariates. For example, the Cox-Time model’s prediction error is under 3% using the proposed extended patients’ covariates versus over 32% using the patients’ time-fixed covariates, and the DeepSurv model’s prediction error is under 4% when we used extended patients’ covariates versus over 32% using the time-fixed covariates.

### 3.2. Predicited Survival Varies by Patient Covariates

**Figure 3:**
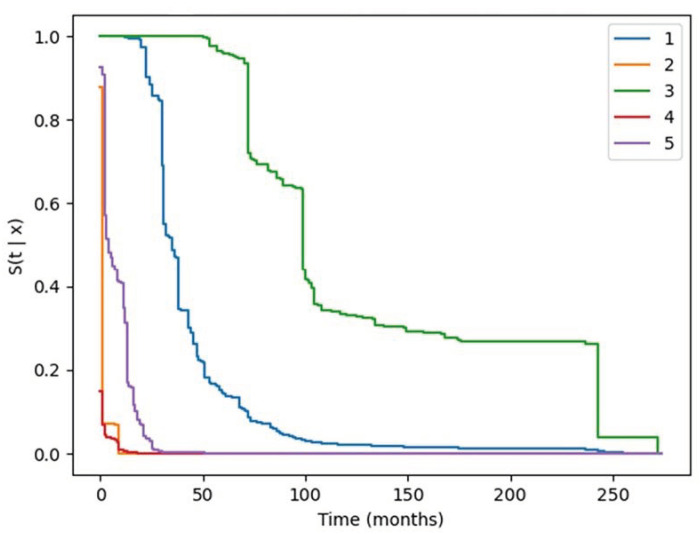
Five hypothetical patient predicted survival curves. No patient details are provided; our DUA with the NCI SEER-Medicare does not allow us to include specific patient-identifying information.

The median of the five hypothetical patient predicted survival curves ranges from less than 1 month to 100 months (Figure 3). The predictive models utilized the unique circumstances created by the interaction of the individual patient, cancer, and treatment variables to generate the individual patient’s predicted survival curves. We cannot provide patient details because our DUA with the NCI SEER-Medicare does not allow us to include specific patient-identifying information.

## 4. Discussion

Our results represent two significant achievements in the predictive modeling of patient outcomes. The first achievement presents a highly significant improvement in the prediction performance of DeepSurv, DeepHit, Nnet-survival, and Cox-Time deep learning models. The models handle time-varying covariates together with time-fixed covariates from the time of diagnosis and prior visits. Using the four deep-learning models, our approach lowered the prediction error rate from 28–38% to 2–12%. The lower values of the IBS, which is the mean square error of prediction, indicated better predictive performance. We estimated the prediction error by accounting for censoring through the cause-specific Concordance Index.

The second achievement is the application of the models to generate individual patients’ predictive survival curves based on their unique patient, cancer, and treatment features. This will permit predictive modeling in the myriads of scenarios encountered with individual patients on likelihood of survival, AEs and impact of progressive therapy. Evidence for guiding therapy after multiple lines of treatment-progression in stage IV BC does not exist in most scenarios and many obstacles prevent development of such clinical trials. The event pattern-based representation of association relationships will enable us to perform reasoning and what-if analysis using temporal logic-based approaches, e.g., treating a patient who has progressed after multiple lines of systemic therapy and selecting the optimum treatment type most likely to prolong survival in the specific scenario generated from the conglomeration of the unique patient, cancer and treatment history variables. Outcomes from these models include the first demonstration that the occurrence of certain AE categories has a negative impact on survival [65]. In that light, the models will inform patients and treating physicians of the most efficacious next line of treatment to prolong survival or of the likelihood of experiencing primarily adverse events with a minimal likelihood of prolonging survival. The Concordance Index and the integrated Brier scores validate the accuracy of the predicted survival curves of individual patients. Prospective studies integrating clinical and genomic data may identify unique clinicogenomic features of metastatic BC patients who can achieve durable disease control without prolonged chemotherapy [47].

As part of our future work, we plan to acquire the Medicaid Analytic eXtract (MAX) dataset and use it to validate the models’ performance and help enhance the generalizability of our results [88]. Our planned follow-up work with the MAX dataset, which has younger patients that are more representative of the population, and also includes added cancer features, including the 21-gene prognostic profile, will address a limitation of the study, which pertains to the higher mean age of the Medicare population that may impact their survival [67].

In our research, we sorted the AEs and treatment codes into general related categories to enable analysis. Thus, the impact of treatments and adverse events that we analyzed may vary among individual drugs or AEs collated into single categories. The AEs we considered as time-varying events are not graded in the SEER-Medicare data as minor or moderate vs. severe. Our future investigations based on data-driven hypotheses generated from this work will include a classification of non-severe and severe AEs, with the latter assigned to AEs associated with emergency department or hospital admissions or death.

## 5. Conclusions

These investigations demonstrate a significant improvement of the deep learning models DeepSurv, DeepHit, Nnet-survival, and Cox-Time. These models outperform the Cox proportional hazard model in survival analysis with the proposed extended patients’ covariates compared with that of the patients’ time-fixed covariates, reducing the error rate of Concordance Indices from 28–38% to 2–12%. The project also developed individual patients’ predictive survival curves based on their unique patient, cancer and treatment and adverse event variables. The models provide a unique tool for medical caregivers to generate realistic survival probabilities for individual patients with stage IV BC given their unique, specific circumstances. Using the model, caregivers will have the guidance to select treatments for individual patient and cancer circumstances that will extend their survival to maximal durations reported for the particular cancer histology, stage, and molecular characteristic, or avoid treatment when the only effects will be adverse events. It will be a highly useful adjunct to the clinical decision-making in individual patients guiding the potential next line of therapy for stage IV BC.

## Figures and Tables

**Table 1:** Population characteristics.

Number of Entries	Number of Patients	Age ± SD	Comorbidity Index ± SD
1,880,153	14,312	76.0 ± 7.5	1.6 ± 2.6

**Table 2:** Hyperparameters.

Hyperparameter	Type	Range
Batch size	Categorical	[32, 64, 128, 256, 512]
Epochs	Categorical	[100, 200, 300, 500]
Dropout rate	Categorical	[0.001, 0.01, 0.1, 0.2, 0.3, 0.4, 0.5, 0.6, 0.7, 0.8]
Number of layers	Integer values	[2, 5]
Number of nodes	Categorical	[32, 64, 128, 256, 512]
Alpha	Categorical	[0.0, 0.001, 0.1, 0.2, 0.5, 0.8, 0.9, 0.99, 1.0]
Sigma	Categorical	[0.01, 0.1, 0.25, 0.5, 1.0, 10, 100]
Learning rate	Continuous	[0.0001, 0.1]

**Table 3:** Stage IV concordance indices.

Model	Time-Dependent Concordance		Integrated Brier Score	
	SM_Time-Fixed Patients’ Covariates	SM_Time-Fixed and Varying Patients’ Covariates	SM_Time-Fixed Patients’ Covariates	SM_Time-Fixed and Varying Patients’ Covariates
Cox-Time	0.680 ± 0.006	0.977 ± 0.005	0.069 ± 0.0046	0.008 ± 0.001
DeepHit	0.719 ± 0.008	0.877 ± 0.004	0.060 ± 0.005	0.014 ± 0.004
DeepSurv	0.678 ± 0.005	0.963 ± 0.118	0.042 ± 0.0004	0.118 ± 0.120
Nnet-Survival (Logistic Hazard)	0.619 ± 0.001	0.892 ± 0.008	0.045 ± 0.003	0.007 ± 0.002

## Data Availability

Original data were obtained from SEER-Medicare dataset under a two-tiered review process. SEER-Medicare data are available to investigators upon review.
